# MicroRNA deregulation and pathway alterations in nasopharyngeal carcinoma

**DOI:** 10.1038/sj.bjc.6604948

**Published:** 2009-03-17

**Authors:** H-C Chen, G-H Chen, Y-H Chen, W-L Liao, C-Y Liu, K-P Chang, Y-S Chang, S-J Chen

**Affiliations:** 1 Genomic Core Laboratory, Molecular Medicine Research Center, Chang Gung University, Taiwan, Republic of China; 2 Department of Otolaryngology, Chang Gung Memorial Hospital at Lin-Kou, Taoyuan, Taiwan, Republic of China

**Keywords:** microRNA, target, pathway, nasopharyngeal carcinoma

## Abstract

MicroRNAs (miRNAs) are a family of small non-coding RNA molecules of about 20–23 nucleotides in length, which negatively regulate protein-coding genes at post-transcriptional level. Using a stem-loop real-time-PCR method, we quantified the expression levels of 270 human miRNAs in 13 nasopharyngeal carcinoma (NPC) samples and 9 adjacent normal tissues, and identified 35 miRNAs whose expression levels were significantly altered in NPC samples. Several known oncogenic miRNAs, including miR-17-92 cluster and miR-155, are among the miRNAs upregulated in NPC. Tumour suppressive miRNAs, including miR-34 family, miR-143, and miR-145, are significantly downregulated in NPC. To explore the roles of these dysregulated miRNAs in the pathogenesis of NPC, a computational analysis was performed to predict the pathways collectively targeted by the 22 significantly downregulated miRNAs. Several biological pathways that are well characterised in cancer are significantly targeted by the downregulated miRNAs. These pathways include TGF-Wnt pathways, G1-S cell cycle progression, VEGF signalling pathway, apoptosis and survival pathways, and IP3 signalling pathways. Expression levels of several predicted target genes in G1-S progression and VEGF signalling pathways were elevated in NPC tissues and showed inverse correlation with the down-modulated miRNAs. These results indicate that these downregulated miRNAs coordinately regulate several oncogenic pathways in NPC.

MicroRNAs (miRNAs) are short non-coding RNA molecules involved in post-transcriptional gene regulation (Ambros, 2004; Bartel, 2004). In animals, miRNAs control the expression of target genes by inhibiting translation or degradating target mRNAs through binding to their 3′UTR (Bartel, 2004). MicroRNAs have been found to regulate genes involved in diverse biological functions, including development, differentiation, proliferation, and apoptosis (Ambros, 2004; Croce and Calin, 2005; Bushati and Cohen, 2007).

Cumulative evidence suggests that miRNAs have major functions in the pathogenesis of tumour. Approximately 50% of miRNAs are localised in cancer-associated genome regions (Calin *et al*, 2004; Sevignani *et al*, 2007). Biological characterisation also identified several miRNAs function as tumour suppressors or oncogenes (Chen, 2005; Croce, 2008). Large scale profiling revealed a global alteration of miRNA expression patterns in human cancers (Lu *et al*, 2005; Murakami *et al*, 2006; Kida and Han, 2008). Recently, distinct miRNA expression signatures have been proposed as diagnostic and prognostic markers for various types of human cancer (Yanaihara *et al*, 2006; Schetter *et al*, 2008; Yu *et al*, 2008).

Although the biological effects of many miRNAs have been characterised individually (Cimmino *et al*, 2005; Raver-Shapira *et al*, 2007; Asangani *et al*, 2008), the impact of multiple miRNA dysregulation on cellular functions and their roles in tumour progression remain largely unknown. Proteomic and microarray data reveal that although each miRNA may regulate up to hundreds of genes, their effect on individual gene is moderate at best (Lim *et al*, 2005; Baek *et al*, 2008; Selbach *et al*, 2008). Recent studies suggest that multiple miRNAs may work in concert to regulate related targets in a common pathway (Cloonan *et al*, 2008; Liu *et al*, 2008). Therefore, pathway analysis, rather than individual target gene characterisation, may provide a better solution to evaluate the biological consequences of global miRNA dysregulation.

To link the miRNA profiling data with biological functions, Gusev *et al* (2007) has developed a strategy, which calculates the functions and pathways collectively regulated by the coexpressed miRNA on the basis of computationally predicted targets. As all target prediction algorithms generate certain fraction of false positives, these false positives may significantly reduce the data reliability when targets predicted for multiple miRNAs were combined to calculate the functional pathways. Recent microarray and proteomic data have provided valuable insights on target prediction (Grimson *et al*, 2007). With these refinements implemented in miRNA target prediction, the results of pathway enrichment analysis on the basis of the coregulated targets should provide a good insight into the functional role of dysregulated miRNAs.

In this study, we used both experimental and computational approaches to assess the functional impact of miRNA dysregulation in nasopharyngeal carcinoma (NPC). Differentially expressed miRNAs were identified by profiling 270 human miRNAs in clinical samples. Target prediction followed by pathway enrichment analysis was conducted to identify the functional pathways specifically regulated by the down-modulated miRNAs. Two modifications were introduced to increase the confidence for target prediction and enhance the specificity for pathway analysis. The identified specific pathways were validated by the expression data and in good agreement with earlier reported pathogenesis in NPC.

## Materials and methods

### Tissue RNA preparation

Samples of NPC tissue and adjacent normal nasopharynx tissue were obtained from patients undergoing surgery and were frozen immediately after surgical resection. Collection and distribution of tissue specimens were performed in accordance with the Institutional Regulation Board of Chang Gung Memorial Hospital, Taiwan. Total RNA was prepared using TRIzol reagent (InVitrogen, Carlsbad, CA, USA) according to the manufacturer's protocol. The concentration of RNA was quantified using a NanoDrop Spectrophotometer. The RNA integrity was evaluated by Agilent 2100 BioAnalyzer (Agilent Technologies, Palo Alto, CA, USA). RNAs with an RNA integrity number (RIN) >7.5 were used for miRNA and mRNA quantifications.

### Reverse transcription (RT)

For miRNA quantification, a pulsed RT reaction, as described by Chen *et al* (2005), was performed to convert all miRNAs into corresponding cDNAs in one RT reaction. Briefly, 10-*μ*l reaction mixture containing miRNA-specific stem-loop RT primers (final 2 nM each), 500 *μ*M dNTP, 0.5 *μ*l Superscript III (InVitrogen), and 1 *μ*g total RNA were used for the RT reaction. The pulsed RT reaction was performed as follows: 16°C for 30 min, followed by 50 cycles at 20°C for 30 s, 42°C for 30 s, and 50°C for 1 s. To quantify mRNA transcripts, 1 *μ*g of total RNA was converted into cDNA using a random hexamer as primer in a 10-*μ*l reaction with the following conditions: 16°C for 30 min, followed by 42°C for 90 min. Reverse transcription products were diluted 20-folds before using for miRNA and mRNA Q-PCR reactions.

### Quantitative RT–(RT-qPCR) PCR

For miRNA quantification, 1 *μ*l of diluted RT product was used as template for a 10 *μ*l PCR. Briefly, 1 × SYBR Master Mix (Applied Biosystem, Foster City, CA, USA), 200 nM miRNA-specific forward primer, and 200 nM universal reverse primer were used for each PCR reaction. The condition for Q-PCR is 95°C for 10 min, followed by 40 cycles of 95°C for 15 s and 63°C for 32 s, and a dissociation stage. Endpoint reaction products were analysed on a 10% polyacrylamide gel stained with ethidium bromide to discriminate the correct amplification product (57–60 bp) and the potential primer dimmers (<44 bp). For mRNA quantification, the following PCR conditions were used: 95°C for 10 min, followed by 45 cycles of 95°C for 15 s and 60°C for 1 min, and a dissociation stage. All Q-PCR reactions were performed on an ABI Prism 7500 Fast Real-Time PCR system (Foster City, CA, USA).

### Data analysis

The threshold cycle (*C*_t_) is defined as the cycle number at which the change of fluorescence intensity crosses the threshold of 0.2. Of the 270 miRNAs evaluated, 47 showed the expression level below the detection limit (*C*_t_>35) in more than 70% of samples, and were excluded from the analysis. For the remaining 223 miRNAs, raw *C*_t_ data were converted to 39 – *C*_t_ normalised by global median normalisation before further analysis. For mRNA expression, the average *C*_t_ of *β*-2-microglobulin, actin, and GAPDH was subtracted from the raw *C*_t_ value to obtain *δ*-*C*_t_ (d*C*_t_). The experimentally normalised d*C*_t_ values were converted to 39 – *C*_t_ used to analyse the expression level of human mRNA transcripts.

Statistical analyses used for miRNA and mRNA expression data including two-sample *t*-tests (two-tailed), paired-sample *t*-test (two-tailed), Mann–Whitney test, principle component analysis, Pearson's correlation analysis, and hierarchical clustering were performed with Partek Genomics Suite (version 6.3, St Louis, MO, USA). Pathway enrichment analysis was performed using the MetaCore database (GeneGO, St Joseph, MI, USA). *P*-values for pathway enrichment analysis were calculated using the formula for hypergeometric distribution and reflects the probability for a pathway to arise by chance.

## Results

### Identification of differentially expressed miRNA in human NPC

The roles of miRNA dysregulation in the pathogenesis of NPC have not been well explored. To identify miRNAs differentially expressed in NPC, we analysed the expression levels of 270 human miRNAs in 13 NPC and 9 normal nasopharyngeal tissues. These samples include seven pairs of NPC and their adjacent normal tissues from same patients. MicroRNA profiling was performed using a stem-loop RT–qPCR method as described by Chen *et al* (2005). The stem-loop RT–qPCR assay has been proven to offer high sensitivity and specificity for the quantification of mature miRNAs. The expression data of 47 miRNAs were eliminated from further analysis because of their low abundance in all samples tested (*C*_t_>35). Expression levels of the remaining 223 miRNAs were expressed as 39 – *C*_t_ after global median normalisation.

**Figure 1:**
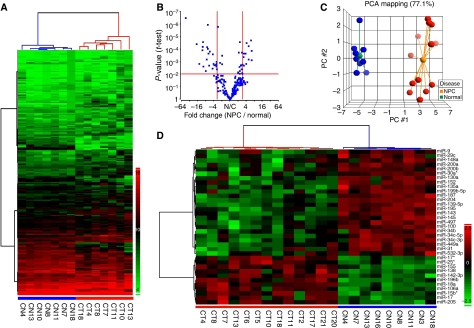
MicroRNA (miRNA) expression patterns distinguish normal from NPC tissues. (**A**) Unsupervised hierarchical clustering of 223 miRNAs in seven normal (blue)–NPC (red) paired tissues. The hierarchical clustering was performed using squared Euclidean as distance measure and Ward's method for linkage analysis. MicroRNA levels were expressed as 39 – *C*_t_ after global median normalisation. (**B**) Selection of miRNAs differentially expressed in seven paired normal–NPC tissues. Differentially expressed miRNAs were selected based on *t*-test (*P*<0.01) and median fold change (⩾3-fold). (**C**) Principle component analysis using the expression levels of 35 miRNAs in 9 normal (blue) and 13 NPC (red) samples. (**D**) Unsupervised hierarchical clustering of 35 differentially expressed miRNAs in normal (blue) and NPC (red) samples. The hierarchical clustering was performed using Pearson's dissimilarity as distance measure and Ward's method for linkage analysis. MicroRNA levels were expressed after standardisation.

Unsupervised hierarchical clustering analysis using expression levels of all 223 detectable miRNAs in the seven paired normal–NPC tissues generated a tree with normal and NPC samples clearly separated into two groups (Figure 1A). To identify differentially expressed miRNAs in normal and NPC tissues, two statistical tests (two-sample *t*-test and Mann–Whitney rank test) were performed. Expression of 35 miRNAs was significantly altered in NPC samples (fold change: ⩾3; false discovery rate: <0.05; Figure 1B). Among them, 11 miRNAs, including miR-196b, miR-138, miR-155, miR-142-3p, and miR-18a, were significantly upmodulated and 24 miRNAs, including miR-204, miR-449a, miR-34c-3p, miR-143, and miR-145, were down-modulated in NPC samples. Complete list of differentially expressed miRNAs and their chromosome location are shown in Table 1. Similar results were obtained when the miRNA expression levels were normalised using the geometric mean of two reference miRNAs, miR-103 and miR-191, as suggested by Peltier and Latham (2008; Supplementary Figure S1 and Supplementary Table S1).

**Figure 2:**
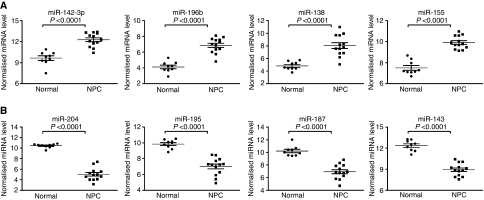
Significantly modulated MicroRNAs (miRNAs) in 9 normal and 13 NPC tissues. (**A**) Expression levels of four miRNAs significantly upregulated in NPC tissues. (**B**) Expression levels of four miRNAs significantly downregulated in NPC tissues. Expression levels of miRNAs were expressed as 39 – *C*_t_ after normalisation. *P*-value for each miRNA was calculated using two-tailed *t*-test.

To test whether the differentially expressed miRNAs selected from paired samples show similar trend of alteration in unpaired samples, we included two additional normal and six additional NPC samples for the principal component analysis (PCA). As shown in Figure 1C, the PCA analysis showed a complete segregation between the 9 normal and 13 NPC samples. Unsupervised hierarchical clustering with the 35 altered miRNAs showed a clear separation between NPC and normal samples. Expression levels of four most significantly upregulated miRNAs and four most significantly down-modulated miRNAs in NPC tissues were shown in Figure 2A and B. The global alteration in miRNA expression pattern observed in our study is similar to earlier reports on other human cancers (Lu *et al*, 2005; Muralidhar *et al*, 2007).

### *In silico* analysis of pathways specifically targeted by down-modulated miRNAs

**Figure 3:**
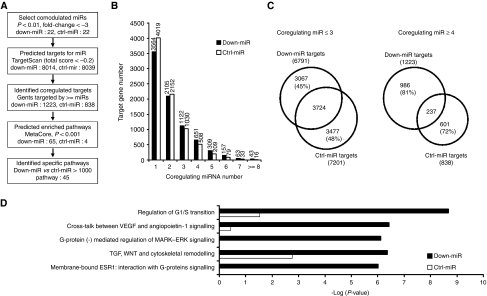
Analysis of collective regulated pathways. (**A**) Flow diagram depicting the process to identify the coregulated targets and specifically enriched pathways. (**B**) Distribution of predicted targets regulated by down-miR and ctrl-miR. (**C**) Distribution of cumulative target number coregulated by multiple microRNAs. (**D**) Pathways significantly and specifically enriched by down-miR targets.

The profiling analysis indicated that a large number of miRNAs are down-modulated in NPC tissues. As miRNAs are negative regulators of protein-coding genes, down-modulation of these miRNAs are expected to cause an upregulation of their target genes and alterations of the associated cellular pathways in NPC tissues. To estimate the overall effect of these down-modulated miRNAs on cellular functions, we adopted a two-stage approach as depicted in Figure 3A. The first stage was designed to identify target genes coregulated by these down-modulated miRNAs. The second stage performed a pathway enrichment analysis using the coregulated targets to identify cellular functions specifically regulated by these down-modulated miRNAs.

Most computational algorithms predict miRNA targets on the basis of sequence complementarity and/or thermostability (Lewis *et al*, 2003; John *et al*, 2004). However, recent studies indicated that many additional factors, such as local AU content and site position, can significantly affect the target site efficacy (Grimson *et al*, 2007). The overall target efficacy can be expressed by a context score. The correlation between context score and site efficacy has been validated in both mRNA and protein levels (Baek *et al*, 2008). Therefore, the concept of context score was implemented to select high confidence targets for the down-modulated miRNAs. The context score threshold for high efficacy target was set at −0.2.

To identify coregulated targets for the down-modulated miRNAs, we retrieved all target genes listed in the TargetScan database (http://www.targetscan.org/; Lewis *et al*, 2003), which includes targets for 456 miRNA families predicted by seed sequence complementarity. Low confidence targets were eliminated by filtering out targets with total context score greater than −0.2. Two miRNAs, miR-34c-3p, and miR-30a^*^ were not present in the TargetScan database, and therefore were excluded from further analysis. The 22 down-modulated miRNAs (‘down-miR’) were used for target prediction and pathway analysis. To enhance the specificity of the target and pathway analysis, a control set of 22 miRNAs (‘ctrl-miR’) was included in the target prediction and pathway analysis. These 22 miRNAs showed constant expression level in normal and NPC tissues and shared no sequence homology in their seed regions with the 22 down-modulated miRNAs. The seed sequence and the number of unique target predicted for individual miRNA were listed in Table 2.

The number of total targets predicted for down-miR and ctrl-miR was 8014 and 8039, respectively. Distribution of predicted targets *vs* coregulating miRNAs for down-miR and ctrl-miR was shown in Figure 3B. Within each target group, approximately half of the predicted targets were recognised by single miRNA (44% of down-miR targets and 50% of the ctrl-miR targets). A majority of targets were recognised by three or fewer miRNAs (85% of down-miR targets and 90% of ctrl-miR targets). Between the two target groups, more than 55% of predicted targets were shared by both groups when the number of coregulating miRNA was equal or less than three. However, when the number of coregulating miRNA was equal or more than four, the fraction of overlapping targets decreased to less than 20%. These results suggested that genes coregulated by four or more miRNAs provided a better distinction between the two target groups and were more suitable for pathway-enrichment analysis.

The number of targets regulated by four or more miRNAs was 1223 and 838 for down-miR and ctrl-miR, respectively. These two target groups were selected and uploaded into MetaCore for pathway enrichment analysis. Statistically enriched pathways were identified using a threshold *P*-value of 0.001. Sixty-five pathways were found to be statistically enriched with the down-miR targets, whereas only four pathways were enriched with the ctrl-miR targets. For each pathway, we calculated the ratio of *P*-values between down-miR and ctrl-miR to determine the specificity. With a *P*-value ratio >1000 as the criteria, we identified 45 pathways specifically enriched by down-miR targets. These specifically targeted pathways include many pathways involved in tumour pathogenesis, such as TGF-Wnt pathways, G1-S cell cycle progression, VEGF signalling pathway, apoptosis and survival pathways, and IP3 signalling pathways. The five most specifically targeted pathways were shown in Figure 3D. Table 3 listed the specifically targeted pathways grouped by their cellular functions.

### Validation of genes specifically targeted by the downregulated miRNAs

**Figure 4:**
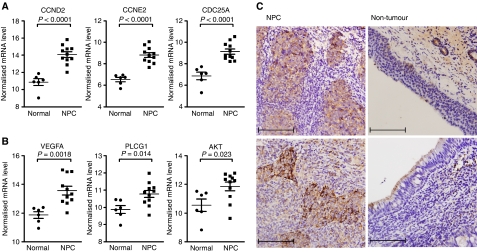
The upregulation of microRNA (miRNA) coregulated targets in NPC. (**A**) Quantification of coregulated targets in growth factor-regulated G1-S cell cycle pathway : cyclin D2 (CCND2), cyclin E2 (CCNE2), and CDC25A. (**B**) Quantification of coregulated targets involved in VEGF pathway: VEGFA, PLCG1 (phospholipase C-*γ*1), and AKT. Expression level of each gene is expressed as 39 – *C*_t_ after normalised to three internal controls. (**C**) Specimens of NPC tissues were stained with the anti-CCNE2 antibody. Shown here are two representative cases containing tumour cells with positive CCNE2 staining (left panels), as well as non-tumour epithelial cells with negative CCNE2 staining (right panels). Original magnification: × 400; Bar, 100 *μ*m.

Regulation of G1/S transition and cross-talk between VEGF and angiopoietin-1 signalling are the two most significantly pathways targeted by down-miR. To validate the effect of down-miR on these two pathways in NPC, we selected three predicted target genes from each pathway and compared their expression levels in normal and NPC samples (Table 4). Real-time RT–PCR analysis revealed that the expression levels of all six predicted targets, including cyclin D2 (CCND2), cyclin E2 (CCNE2), CDC25A, VEGFA, phospholipase C-*γ*1 (PLCG1), and AKT3, were significantly elevated in NPC tissues (Figure 4A and B). We also examined the protein levels of CCNE2 in NPC tissue sections by immunohistochemical staining to identify which cell type in the tumour mass express cyclin E2 protein. Two representative cases of cyclin E2 staining are shown in Figure 4C. Strong positive cyclin E2 staining was detected in tumour cells (left panels), but not in paired normal nasopharyngeal epithelium (right panels). This result confirmed that the predicted targets of down-miR are upregulated in NPC.

**Figure 5:**
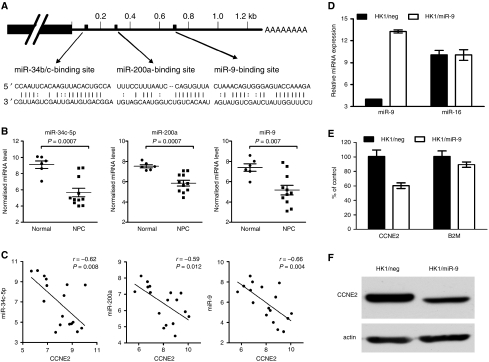
Inverse correlation between cyclin E2 expression with miR-34c, miR-200a, and miR-9 in NPC. (**A**) Predicted binding sites for miR-34c-5p, miR-200a, and miR-9 on the 3′UTR of cyclin E2. (**B**) Expression levels of miR-34c-5p, miR-200a, and miR-9 in normal and NPC tissues. (**C**) Correlation analysis between cyclin E2 and three microRNAs. Pearson's correlation coefficient and *P*-value for individual analysis are shown in the inserts. (**D**) Quantification of miR-9 in HK1 cells after transduction with either negative control lentivirus (HK1/neg) or lentivirus encoding miR-9 (HK1/miR-9). Levels of miR-9 and miR-16 were expressed as 39 – *C*_t_. (**E**) Quantification of CCNE2 and *β*-2-microglobulin (B2M) transcripts in HK1/neg and HK1/miR-9 cells. Results are mean±s.d. of three independent experiments. A significant reduction in CCNE2 level was observed after miR-9 expression (HK1/neg *vs* HK1/miR-9, CCNE2: *P*=0.014, B2M: *P*=0.258). (**F**) Reduced expression of CCNE2 protein in HK1/miR-9 cells.

The 3′UTR of cyclin E2 contains binding sites for many different miRNAs. Six of them (miR-34c-5p, miR-449, miR-200a, miR-200b, miR-195, and miR-9) were down-modulated in NPC (Figure 5A and B). We further analysed the expression correlation between three of the down-modulated miRNAs and cyclin E2 in 16 samples, including 10 NPC and 6 normal nasopharyngeal tissues. Expression of all three miRNAs showed significant inverse correlation (Pearson's correlation coefficient <−0.5, *P*<0.05) with the expression level of cyclin E2 (Figure 5C). These results indicated that the down-modulated miRNAs cooperatively upregulated critical targets in the G1/S transition pathway. To determine whether the expression level of CCNE2 is indeed regulated by down-modulated miRNAs, we examined the mRNA and protein level of CCNE2 in HK1 cells overexpressing one of the coregulating miRNA, miR-9 (Figure 5D). As shown in Figure 5E and F, HK1/miR-9 cells showed a significant decrease in CCNE2 mRNA and protein levels, indicating that CCNE2 is a direct target of miR-9.

## Discussion

This study used a stem-loop RT–PCR method to quantify the expression levels of 270 miRNAs in NPC tissues. Using a three-fold change as the cutoff, we identified 35 miRNAs whose expression levels were significantly altered in NPC tissues. Unsupervised hierarchical clustering using these differentially expressed miRNAs clearly segregated NPC tissues from the normal samples. Among the differentially expressed miRNAs identified in this study, many are known oncogenic or tumour-suppressive miRNAs previously shown to be dysregulated in other cancer types (Chen, 2005; Croce, 2008). Our results provided further support for using miRNA expression patterns as diagnostic markers.

The profiling analysis identified a group of miRNAs whose expression was significantly down-modulated in NPC. To assess the global impact of these down-modulated miRNAs, we conducted a high stringency target prediction to identify potential target genes, which were coregulated by multiple down-modulated miRNAs, and then analysed the cellular pathways, which were specifically enriched with these coregulated genes. Results from the pathway enrichment analysis suggested that these down-modulated miRNAs selectively target signalling cascades involved in cell cycle regulation, cell survival and apoptosis, and cytoskeleton remodelling. Quantification of target genes in clinical samples confirmed the elevated expression of several coregulated targets, and revealed a significant inverse correlation between the down-modulated miRNAs and their targets. These results indicated that the target prediction combined with pathway enrichment analysis is an effective approach in investigating the influence of multiple miRNAs.

Recently, Sengupta *et al* (2008) identified eight miRNAs differentially expressed in NPC. Three of the most significantly altered miRNAs, including miR-29c, miR-34b, and miR-34c, also showed significantly reduced expression in our NPC samples. However, the remaining five miRNAs identified in their study did not show significant alteration in the current study. The discrepancy could be due to the difference in sample preparation or difference between microarray and RT–PCR assay.

Expression of many oncogenic miRNAs, including miR-155, miR-106a, and three miRNAs from the miR-17-92 cluster, were found significantly elevated in NPC. The miR-155 upregulates the NF-*κ*B signalling pathway and is highly expressed in many haematological malignancies and solid tumours (Rai *et al*, 2008). MicroRNAs from the miR-17-92 cluster suppress multiple proliferation inhibitors as well as proapoptotic targets, and promote tumour formation (Matsubara *et al*, 2007; Cloonan *et al*, 2008). The upregulation of the miR-17-92 cluster is noted in leukaemia, thyroid cancer, and lung cancer (Hayashita *et al*, 2005; Venturini *et al*, 2007; Takakura *et al*, 2008).

The down-modulated mRNAs include miR-34b/miR-34c cluster, the miR-143/miR-145 cluster, and the miR-195/miR-497 cluster. Both miR-34b and miR-34c are downstream components of the p53 tumour suppressor network (Corney *et al*, 2007; Tarasov *et al*, 2007). Induction of miR-34b and miR-34c leads to apoptosis or cellular senescence, whereas reduced miR-34b/c expression attenuates p53-mediated cell death (Tarasov *et al*, 2007; Welch *et al*, 2007). Reduced expression of miR-143 and miR-145 has been documented in cervical cancer, colon cancer, and B-cell malignancies (Michael *et al*, 2003; Akao *et al*, 2007; Wang *et al*, 2008). Forced expression of miR-143 and miR-145 inhibit cell growth in cervical cancer cells (Wang *et al*, 2008). Although the biological function of miR-195 and miR-497 has not been characterised, reduced expression of miR-195 and miR-497 on the 17p13 locus has been documented in primary peritoneal carcinoma (Flavin *et al*, 2008).

In addition to the commonly observed miRNAs, this study also identified several dysregulated miRNAs whose aberrant expression has not been reported before. For example, miR-25^*^ was significantly elevated in NPC and miR-532, and miR-199b-5p were down-modulated. MiR-449 shared the same seed sequence with miR-34c and was found down-modulated in our study. Similarly, miR-152 shared the same seed sequence with miR-148a, and both miRNAs were down-modulated in NPC.

Recently, two studies using microarray and proteomic approach clearly showed that a single miRNA can regulate the expression of hundreds of target genes (Baek *et al*, 2008; Selbach *et al*, 2008). The widespread repression can be detected in both mRNA and protein levels, but the degree of repression for individual target gene is usually very mild. On the other hand, miRNA profiling analysis typically uncovered aberrant expression of multiple miRNAs in the diseased tissues (Lu *et al*, 2005). How to assess the biological consequence of miRNA dysregulation has become a major challenge. To overcome this difficulty, Gusev *et al* (2007) used a computational approach to calculate the global pattern of cellular functions and pathways affected by the coexpressed miRNAs.

In this study, we adopted the systems biology-based approach to investigate the global impact of differentially expressed miRNAs in the pathogenesis of NPC. Similar to the strategy described by Gusev *et al* (2007), we analysed the major signalling pathways that are most likely to be affected by the coexpressed miRNAs. Two modifications were introduced to enhance our confidence in target prediction and pathway analysis.

To enhance the accuracy for miRNA target prediction, we implemented the ‘context score’ concept during the target prediction process. Only genes with a total context score less than −0.2 were included in our target pool. The concept of context score was first proposed by Grimson *et al* (2007) to evaluate the site efficacy of miRNA target using microarray data. The ‘context score’ combined the complementarity of seed sequence, local AU content, site position, and additional base pairing at the 3′ site of miRNA to evaluate the site efficacy between miRNAs and their targets. This concept was further supported by the data from recent proteomic studies (Baek *et al*, 2008).

To enhance the specificity of pathway enrichment analysis, we selected a set of control miRNAs, which were not altered in NPC, and conducted the target prediction and pathway enrichment analysis in parallel. The probability of a gene targeted by multiple miRNAs increases as the length of its 3′UTR increases. This potential bias introduced by genes with longer 3′UTR can be minimised by comparing it with the targets coregulated by the control miRNAs. The pathway specificity was calculated by comparing the *P*-values between these two target sets. This strategy effectively addressed the complexity that arise from the coexpressed miRNAs and coregulated targets.

Our studies revealed that the targets coregulated by the down-modulated miRNA are specifically enriched with genes involved in ‘TGF-WNT and cytoskeletal remodelling’ pathways. Predicted targets include several key regulators of the Wnt pathway, such as WNT1, WNT2, frizzled homologue 3, 7, and 8, AKT, and NF-*κ*B1, as well as multiple extracellular matrix proteins, such as collagen 4A1, 4A4, and fibronectin 1. Studies by Zeng *et al* (2007), using profiling and immunohistochemical stains, shows that the protein levels of these coregulated targets were significantly elevated in NPC tissues, whereas elevated expression of extracellular matrix proteins was shown by Sengupta *et al* (2008) using Q-PCR. The analysis is consistent with the observation that WNT signalling pathway is abnormally regulated in NPC (Shi *et al*, 2006; Zeng *et al*, 2007; Chou *et al*, 2008).

Another pathway preferentially targeted by the down-modulated miRNAs involved growth factor-regulated G1-S cell cycle progression. Several cell cycle regulators, including cyclin D2, cyclin E2, and CDC25A are coregulated by the downregulated miRNAs. Reverse transcriptase PCR analysis confirmed the elevated expression of these target genes in NPC tissues. Correlation analysis further revealed a significant inverse correlation between the level of cyclin E2 and three of its cognate miRNAs, miR-34c, miR-200a, and miR-9. These results suggest that the aberrant cell cycle control in NPC tissues may be tightly linked to the dysregulated expression of miRNAs (Hwang *et al*, 2002; Tao *et al*, 2005; Chou *et al*, 2008).

The third signalling pathway, significantly targeted by down-modulated miRNAs, is the VEGF-regulated angiogenesis. Predicted targets include VEGF-A, PLC-*γ*, and AKT. Earlier studies have shown that VEGF is widely expressed in NPC tissues, and elevated VEGF expression is associated with local recurrence and distal metastasis (Li *et al*, 2008; Pan *et al*, 2008). Current studies revealed that several key regulators on the VEGF-regulated signalling pathway are cotargeted by multiple miRNAs, including miR-135a, miR29c, miR-195, and miR-497. Simultaneous down-modulation of these miRNAs may be responsible for the elevated VEGF signalling in NPC.

Recent studies using an miRNA target reverse screening method showed that miR-16 family triggers a cell cycle arrest by silencing multiple cell cycle regulatory genes simultaneously (Liu *et al*, 2008), rather than the individual target. Similarly, miR-17-5p regulates the cell cycle progression by coordinately suppressing more than 20 genes involved in the G1/S transition (Cloonan *et al*, 2008). It is evident that a better connection between cellular function and miRNA expression requires both computational as well as experimental approach. The context score filter and the use of control gene list significantly increased our confidence for target prediction and pathway analysis. Experimental validation and literature mining confirmed that the expression data detected in clinical samples are consistent with the computational prediction. This study has shown the systems biology approach as a valuable tool to uncover global functional change associated with miRNA dysregulation. Similar approach can be used to assist the functional interpretation of miRNA expression data in other human diseases.

## Figures and Tables

**Table 1:** MicroRNAs significantly altered in NPC tissues

		**Mean expression^a^**			***P*-value**	
**MicroRNA**	**Chromosome**	**Normal**	**NPC**	**Fold change**	***T*-test**	**Mann–Whitney**
*U-regulated*						
miR-196b	7p15	4.04	6.75	6.54	1.45E−04	2.68E−03
miR-138	3p21, 16q13	4.58	7.27	6.45	7.77E−04	4.04E−03
miR-155	21q21	7.50	10.09	6.04	2.05E−05	1.75E−03
miR-142-3p	17q22	9.43	11.73	4.92	6.95E−04	1.75E−03
miR-18a	13q31	5.20	7.50	4.92	6.00E−05	1.75E−03
miR-25^a^	7q22	7.22	9.28	4.18	3.43E−05	1.75E−03
miR-205	1q32	16.01	18.02	4.00	1.51E−03	4.04E−03
miR-106a	Xq26	9.25	11.18	3.80	2.37E−04	4.04E−03
miR-17	13q31	10.67	12.41	3.34	2.08E−03	8.81E−03
miR-15b^a^	3q26	4.68	6.39	3.27	2.38E−04	1.75E−03
miR-17^a^	13q31	5.15	6.84	3.22	4.40E−04	1.75E−03
						
*Downregulated*						
miR-204	9q21	10.37	4.91	−44.15	3.51E−07	1.75E−03
miR-449a	5q11	8.54	4.42	−17.45	4.28E−05	2.68E−03
miR-34c-3p	11q23	11.92	8.15	−13.64	1.55E−03	6.01E−03
miR-187	18q12	10.23	6.80	−10.74	1.26E−04	1.75E−03
miR-145	5q33	16.77	13.39	−10.42	7.03E−05	1.75E−03
miR-143	5q33	12.50	9.17	−10.07	2.89E−05	1.75E−03
miR-34c-5p	11q23	9.30	6.04	−9.63	2.49E−03	8.81E−03
miR-34b	11q23	8.36	5.37	−7.92	5.74E−03	8.81E−03
miR-100	11q24	12.93	10.05	−7.36	6.79E−04	1.75E−03
miR-9	1q22, 5q14, 15q26	7.16	4.34	−7.07	7.34E−05	1.75E−03
miR-139-5p	11q13	11.59	8.83	−6.75	1.18E−06	1.75E−03
miR-195	17p13	9.98	7.26	−6.61	9.96E−05	1.75E−03
miR-148a	7p15	8.13	5.46	−6.36	2.33E−04	2.68E−03
miR-30a^a^	6q13	10.08	7.50	−5.99	2.10E−03	8.81E−03
miR-497	17p13	12.12	9.63	−5.62	1.64E−04	2.68E−03
miR-135a	3p21, 12q23	7.88	5.71	−4.49	3.63E−03	4.04E−03
miR-130a	11q12	9.94	7.84	−4.29	2.77E−04	2.68E−03
miR-31	9p21	12.53	10.51	−4.05	1.50E−03	6.01E−03
miR-199b-5p	9q34	7.67	5.74	−3.83	2.05E−03	6.01E−03
miR-200a	1p36	7.32	5.59	−3.32	1.87E−03	4.04E−03
miR-29c	1q32	7.78	6.09	−3.25	2.53E−03	1.27E−02
miR-152	17q21	10.30	8.65	−3.13	1.04E−03	6.01E−03
miR-200b	1p36	12.01	10.36	−3.13	1.59E−03	1.75E−03
miR-532-3p	Xp11	13.52	11.92	−3.03	4.10E−03	8.81E−03

a Mean expression levels expressed as 39 – *C*_t_.

**Table 2:** MicroRNAs selected for taget analysis

	**Seed sequence**	**Target^a^**		**Seed sequence**	**Target^a^**
Down-miR			Ctrl-miR		
miR-100	ACCCGUA	44	miR-101	ACAGUAC	1063
miR-130a	AGUGCAA	865	miR-103	GCAGCAU	759
miR-135a	AUGGCUU	757	miR-129-3p	AGCCCUU	480
miR-139-5p	CUACAGU	650	miR-140-3p	ACCACAG	857
miR-143	GAGAUGA	736	miR-146a	GAGAACU	640
miR-145	UCCAGUU	956	miR-146b-5p	GAGAACU	642
miR-148a	CAGUGCA	808	miR-186	AAAGAAU	2030
miR-152	CAGUGCA	798	miR-193a-3p	ACUGGCC	423
miR-187	CGUGUCU	85	miR-193b	ACUGGCC	422
miR-195	AGCAGCA	1078	miR-194	GUAACAG	780
miR-199b-5p	CCAGUGU	637	miR-24	GGCUCAG	768
miR-200a	AACACUG	1255	miR-27b	UCACAGU	1162
miR-200b	AAUACUG	1393	miR-299-5p	GGUUUAC	511
miR-204	UCCCUUU	1158	miR-320	AAAGCUG	1017
miR-29c	AGCACCA	722	miR-324-5p	GCAUCCC	341
miR-31	GGCAAGA	647	miR-362-5p	AUCCUUG	493
miR-34b	AAUCACU	618	miR-365	AAUGCCC	560
miR-34c-5p	GGCAGUG	703	miR-483-3p	CACUCCU	458
miR-449a	GGCAGUG	716	miR-500	AAUCCUU	354
miR-497	AGCAGCA	994	miR-516a-5p	UCUCGAG	622
miR-532-5p	AUGCCUU	597	miR-550	GUGCCUG	417
miR-9	CUUUGGU	824	miR-654-3p	AUGUCUG	494

a Targets predicted by the TargetScan using a threshold of total score <−0.2.

**Table 3:** Specifically targeted pathways

		**Gene number**			***P*-value**		
**Category**	**Pathway**	**Total^a^**	**Down-miR**	**Ctrl-miR**	**Down-miR**	**Ctrl-miR**	**Ratio**
Cytoskeleton remodelling	TGF, WNT, and cytoskeletal remodelling	102	17	6	7.16E−08	4.77E−03	6.66E+04
	Cytoskeleton remodelling	87	15	6	2.72E−07	2.16E−03	7.93E+03
	ACM3 and ACM4 in keratinocyte migration	37	7	0	2.51E−04	1.00E+00	3.99E+03
	Role of PKA in cytoskeleton reorganisation	39	8	2	4.90E−05	1.21E−01	2.47E+03
Cell cycle	Regulation of G1/S transition	40	10	3	7.90E−07	2.33E−02	2.95E+04
Development	Cross-talk between VEGF and angiopoietin-1 signalling	26	9	1	1.29E−07	3.33E−01	2.58E+06
	Membrane-bound ESR1: interaction with G-proteins	45	10	0	2.55E−06	1.00E+00	3.93E+05
	IGF-RI signalling	43	10	1	1.63E−06	4.88E−01	3.00E+05
	Ligand-independent activation of ESR1 and ESR2	44	10	1	2.04E−06	4.96E−01	2.43E+05
	G-protein-mediated regulation of MARK–ERK signalling	44	9	0	1.68E−05	1.00E+00	5.94E+04
	A3 receptor signalling	47	9	0	2.95E−05	1.00E+00	3.39E+04
	*β*-Adrenergic receptor signalling through cAMP	52	9	0	6.84E−05	1.00E+00	1.46E+04
	Membrane-bound ESR1: interaction with growth facto	38	8	1	4.02E−05	4.47E−01	1.11E+04
	Dopamine D2 receptor transactivation of EGFR	24	6	0	1.39E−04	1.00E+00	7.22E+03
	Leptin signalling through PI3K-dependent pathway	46	9	2	2.46E−05	1.58E−01	6.42E+03
	Notch activating pathway for NF-*κ*B	25	6	0	1.77E−04	1.00E+00	5.65E+03
	VEGF signalling through VEGFR2 – generic cascades	35	7	1	1.74E−04	4.20E−01	2.41E+03
	VEGF signalling and activation	36	7	1	2.10E−04	4.29E−01	2.05E+03
	A1 receptor signalling	50	8	1	3.07E−04	5.41E−01	1.77E+03
	*δ*-Type opioid receptor-mediated cardioprotecti	37	7	1	2.51E−04	4.38E−01	1.75E+03
	ERBB family signalling	38	7	1	2.98E−04	4.47E−01	1.50E+03
	GDNF family signalling	44	7	0	7.57E−04	1.00E+00	1.32E+03
Signal transduction	AKT signalling	40	9	1	7.34E−06	4.64E−01	6.32E+04
	Calcium signalling	43	9	2	1.38E−05	1.42E−01	1.03E+04
	ERK interactions: inhibition of ERK	35	8	2	2.12E−05	1.01E−01	4.77E+03
	IP3 signalling	49	9	2	4.19E−05	1.74E−01	4.16E+03
	cAMP signalling	36	8	2	2.64E−05	1.06E−01	4.01E+03
	PTEN pathway	45	7	0	8.71E−04	1.00E+00	1.15E+03
Apoptosis and survival	BAD phosphorylation	41	8	1	7.17E−05	4.72E−01	6.59E+03
Transcription	ChREBP regulation pathway	22	6	0	8.15E−05	1.00E+00	1.23E+04
	CREB pathway	42	9	2	1.13E−05	1.37E−01	1.21E+04
	Androgen receptor nuclear signalling	45	7	0	8.71E−04	1.00E+00	1.15E+03
	PPAR pathway	60	9	2	2.15E−04	2.36E−01	1.10E+03
Immune response	MIF – the neuroendocrine–macrophage connector	43	9	1	1.38E−05	4.88E−01	3.54E+04
	PGE2 common pathways	52	9	2	6.84E−05	1.91E−01	2.79E+03
	PIP3 signalling in B lymphocytes	42	8	2	8.59E−05	1.37E−01	1.59E+03
Cell adhesion	Chemokines and adhesion	89	13	5	1.16E−05	1.19E−02	1.02E+03
G-protein signalling	G-protein *β*/*γ* signalling cascades	32	8	0	1.04E−05	1.00E+00	9.63E+04
	G-protein *α*-Q signalling cascades	33	7	1	1.17E−04	4.02E−01	3.42E+03

a Total gene number in the pathway.

**Table 4:** Targets selected for validation

**Target gene**	**Expression level^a^**			**Coregulating miRNAs**	
**Symbol**	**Normal**	**NPC**	***P*-value**	**Down-miR**	**Ctrl-miR**
CCND2	10.87	14.06	<0.0001	miR-200a, 204, 29c, and 497	—
CCNE2	6.53	8.81	<0.0001	miR-195, 200a, 200b, 34c, 449, and 9	—
CDC25A	6.87	9.13	<0.0001	miR-100, 195, 34b, and 497	miR-103 and 27
AKT3	10.55	11.86	0.0232	miR-135a, 139, 195, 29c, 497, and 532	miR-140, 365, and 654
PLCG1	9.88	10.78	0.0135	miR-135a, 200b, 204, and 449	miR-140
VEGFA	11.87	13.57	0.0018	miR-195, 200b, 29c, and 497	miR-186

a Expression levels expressed as 39 – *C*_t_ values after normalisation with *β*-2-microglobulin, actin, and GAPDH.
