# Supplementary material for: MicroRNA deregulation and pathway alterations in nasopharyngeal carcinoma
Source: Br J Cancer. 2009 Mar 17;100(6):1002–11. doi: 10.1038/sj.bjc.6604948 (PMC2661776; doi:10.1038/sj.bjc.6604948)

**Supplementary Material**

**MicroRNA Deregulation and Pathway Alterations in Nasopharyngeal Carcinoma**

^1^**Hua-Chien Chen,** ^1^**Gian-Hung Chen,** ^1^**Yi-Hsuan Chen,** ^1^**Wen-Ling Liao,** ^1^**Chien-Yuan Liu,** ^2^**Kai-Ping Chang,** ^1^**Yu-Sun Chang and** ^1^**Shu-Jen Chen***

**Content**

**Material and Methods page 1**

**Figure legend page 3**

**Figure S1 page 4**

**Table S1 page 5**

**Material and Methods**

**Western blot analysis**

Protein extracts, separated by SDS-PAGE and transferred onto PVDF membranes, were probed with antibodies against CCNE2 (SC-28351, 1:5000, Santa Cruz Biotechnology, Santa Cruz, CA) or actin (MAB1501, 1:5000, Chemicon, Billerica, MA). Proteins of interest were detected with HRP-conjugated sheep anti-mouse IgG antibody (1: 5000, GE Healthcare, Uppsala, Sweden) and visualized with the Pierce ECL Western blotting substrate (Thermo Scientific, Rockford, IL), according to the provided protocol.

**Immunohistochemistry**

Tissue sections were fixed with 10% formaldehyde, embedded in paraffin and cut into 4 mm-thick sections. Staining for CCNE2 was carried out using the Envision-kit (DAKO, Carpinteria, CA). Briefly, the sections were deparaffinized with xylene, dehydrated with ethanol and then heated in 0.01 M citrate buffer (pH 6.0). Endogenous peroxidase activities were inactivated in 3% H2O2 for 10 min at room temperature, and the sections were blocked with 3% normal goat serum in 0.2 M PBS (pH 7.4). Samples were then incubated with anti-CCNE2 at room temperature for 1 hour. Secondary anti-mouse antibody-coated polymer peroxidase complexes (DAKO) were then applied for 30 min at room temperature, followed by treatment with substrate/chromogen (DAKO) and a further incubation for 5–10 min at room temperature. Slides were counterstained with hematoxylin.

**Construction and Transfection of Lentiviral vectors with miR-9**

Double-stranded oligonucleotide encoding miR-9 precursor or negative control (scrambled sequence) were synthesized and inserted into pcDNA6.2-GW/Em-GFP-miR expression vector (InVitrogen, Carlsbad, CA) which contains human CMV promoter. The miR-9 and negative control expression cassette was transferred into the lentiviral expression plasmid (pLenti6/V5-DEST) with the Gateway recombination technology using the pDONR221 vector as an intermediate vector. To generate lentiviral particles, the miR-9 or negative control lentiviral expression vector were co-transfected with packaging vectors (ViraPower packaging Mix, InVitrogen) into HEK293-FT cells using lipofectamine 2000 (InVitrogen). Culture supernatants were harvested on day 3 and used to infect HK1 cells.

**Cell culture**

Human NPC cell line, HK1, was cultured in RPMI-1640 (InVitrogen) with 10% fetal calf serum at 37 degree in a humidified chamber containing 5% CO2. HEK293-FT cells were maintained DMEM (InVitrogen) supplemented with 10% fetal calf serum and 0.5 mg/L G-418.

**Figure Legend**

**Figure S1**. MicroRNA expression patterns distinguish normal from NPC tissues. (A). Unsupervised hierarchical clustering of 223 miRNAs in 7 normal (blue)-NPC (red) paired tissues. The hierarchical clustering was performed using squared Euclidean as distance measure and Ward’s method for linkage analysis. miRNA levels were expressed as 39 – Ct after normalized to the geometric mean of miR-103 and miR-191 (Peltier & Latham, 2008). (B). Selection of miRNAs differentially expressed in 7 paired normal-NPC tissues. Differentially expressed miRNAs were selected based on t-test (p < 0.01) and median fold change (≥ 3 fold). Seven miRNAs showed significant up-regulation and 27 miRNAs showed significant down-modulation in NPC samples. (C). Principle component analysis using the expression levels of 34 miRNAs in 9 normal (blue) and 13 NPC (red) samples. (D). Unsupervised hierarchical clustering of 34 differentially expressed miRNAs in normal (blue) and NPC (red) samples. The hierarchical clustering was performed using Pearson’s dissimilarity as distance measure and Ward’s method for linkage analysis. MiRNA levels were expressed after standardization.

Peltier HJ, Latham GJ (2008) Normalization of microRNA expression levels in quantitative RT-PCR assays: identification of suitable reference RNA targets in normal and cancerous human solid tissues. *RNA* 14: 844-52

**Figure S1**


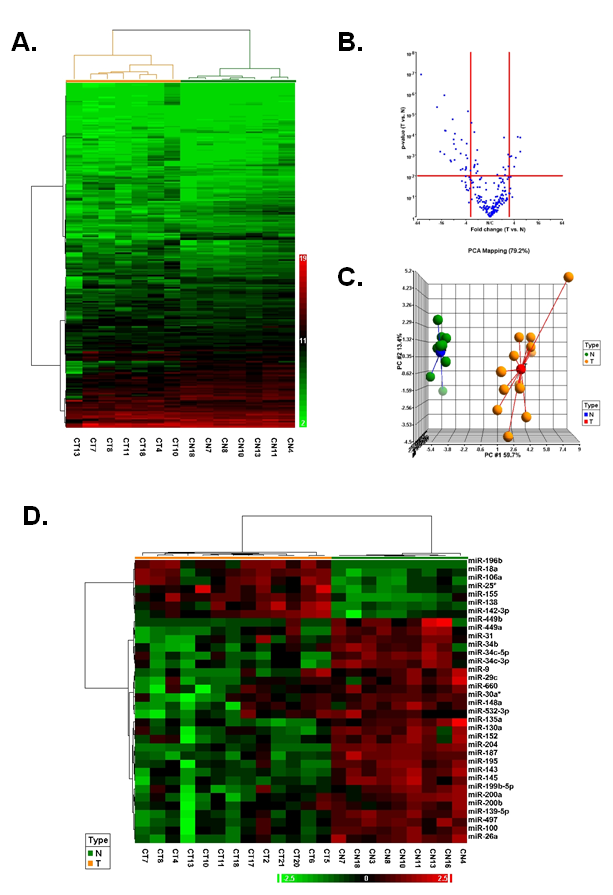

Supplement: Supplementary Material [file 6604948x1.doc]
